# A Paleolithic diet confers higher insulin sensitivity, lower C-reactive protein and lower blood pressure than a cereal-based diet in domestic pigs

**DOI:** 10.1186/1743-7075-3-39

**Published:** 2006-11-02

**Authors:** Tommy Jönsson, Bo Ahrén, Giovanni Pacini, Frank Sundler, Nils Wierup, Stig Steen, Trygve Sjöberg, Martin Ugander, Johan Frostegård, Leif Göransson, Staffan Lindeberg

**Affiliations:** 1 Department of Clinical Sciences, Lund University, Box 117, 221 00 Lund, Sweden; 2 Metabolic Unit, Institute of Biomedical Engineering, National Research Council, Corso Stati Uniti, 4, 35127 Padova, Italy; 3 Department of Experimental Medical Science, Lund University, Box 117, 221 00 Lund, Sweden; 4 Department of Physiological Sciences, Lund University, Box 117, 221 00 Lund, Sweden; 5 Department of Cardiothoracic Surgery, Heart-Lung Division, Lund University, Box 117, 221 00 Lund, Sweden; 6 Department of Clinical Physiology, Lund University, Box 117, 221 00 Lund, Sweden; 7 Department of Medicine, CIM, Karolinska University Hospital, Huddinge, 141 86 Stockholm, Sweden; 8 The Swedish Farmers Supply and Crop Marketing Association, Box 30192, 104 25 Stockholm, Sweden

## Abstract

**Background:**

A Paleolithic diet has been suggested to be more in concordance with human evolutionary legacy than a cereal based diet. This might explain the lower incidence among hunter-gatherers of diseases of affluence such as type 2 diabetes, obesity and cardiovascular disease. The aim of this study was to experimentally study the long-term effect of a Paleolithic diet on risk factors for these diseases in domestic pigs. We examined glucose tolerance, post-challenge insulin response, plasma C-reactive protein and blood pressure after 15 months on Paleolithic diet in comparison with a cereal based swine feed.

**Methods:**

Upon weaning twenty-four piglets were randomly allocated either to cereal based swine feed (Cereal group) or cereal free Paleolithic diet consisting of vegetables, fruit, meat and a small amount of tubers (Paleolithic group). At 17 months of age an intravenous glucose tolerance test was performed and pancreas specimens were collected for immunohistochemistry. Group comparisons of continuous variables were made by use of the t-test. P < 0.05 was chosen for statistical significance. Simple and multivariate correlations were evaluated by use of linear regression analysis.

**Results:**

At the end of the study the Paleolithic group weighed 22% less and had 43% lower subcutaneous fat thickness at mid sternum. No significant difference was seen in fasting glucose between groups. Dynamic insulin sensitivity was significantly higher (p = 0.004) and the insulin response was significantly lower in the Paleolithic group (p = 0.001). The geometric mean of C-reactive protein was 82% lower (p = 0.0007) and intra-arterial diastolic blood pressure was 13% lower in the Paleolithic group (p = 0.007). In evaluations of multivariate correlations, diet emerged as the strongest explanatory variable for the variations in dynamic insulin sensitivity, insulin response, C-reactive protein and diastolic blood pressure when compared to other relevant variables such as weight and subcutaneous fat thickness at mid sternum. There was no obvious immunohistochemical difference in pancreatic islets between the groups, but leukocytes were clearly more frequent in sampled pancreas from the Cereal group.

**Conclusion:**

This study in domestic pigs suggests that a Paleolithic diet conferred higher insulin sensitivity, lower C-reactive protein and lower blood pressure when compared to a cereal based diet.

## Background

Our pre-agricultural, hunter-gatherer human ancestors during the Paleolithic period (the old stone age; 2.5 million – 10,000 years BP) had a diet based on vegetables, fruit, nuts, roots, meat, organ meats and insects [1]. This Paleolithic diet has been suggested to be more in concordance with our evolutionary legacy than a diet based on products associated with agriculture during the Neolithic period (10,000 years BP – present time) such as cereals and milk [2,3]. A diet in less concordance with our evolutionary legacy might confer diseases due to insufficient adaptation [4] possibly explaining the reported lower incidence of diseases of affluence [5], such as type 2 diabetes, obesity and cardiovascular disease among Non-Western ethnic groups with hunter-gatherer lifestyles and diets [6,7]. However, the mechanisms behind this reported lower incidence of diseases of affluence are largely unknown, and commonly discussed dietary factors such as fat (amount and quality) and (cereal) fiber may be of limited importance [8-10]. A possible mechanism might be the potential of Paleolithic diet to reduce risk factors for diseases of affluence, such as disturbed glucose homeostasis, insulin resistance and hypertension [2]. Food restriction induced by the satiating properties of a Paleolithic diet [2] may also play a role. Food restriction, sometimes referred to as dietary restriction or caloric restriction [11], of 20–40% of dietary energy compared to free feeding (*ad libitum*) has been shown to reduce the incidence of several diseases of affluence in mammals including non-human primates, and possibly humans [12,13]. Interestingly, Non-Western ethnic groups with hunter-gatherer lifestyles and diets stay lean and apparently reap health benefits similar to those induced by food restriction despite *ad libitum *availability of food [14].

The aim of this study was to determine experimentally whether a Paleolithic diet on a long-term basis affects risk factors for diseases of affluence in a prospective and randomized setting. This was assessed in domestic swine by examining glucose tolerance, post-challenge insulin response, plasma C-reactive protein and blood pressure after 15 months on a Paleolithic diet in comparison with a cereal based swine feed supplemented with rapeseed oil.

## Methods

### Animals and diet

The animals in this study received humane care in compliance with the "Principles of Laboratory Animal Care" formulated by the National Society for Medical Research and the "Guide for the Care and Use of Laboratory Animals" (National Institutes of Health publication 85-23, revised 1985). The Ethical Committee for Animal Experiments at Lund University approved the study (diary number M 263-02). Twenty-four cross-bred (dam (Swedish Landrace × Yorkshire) × sire Hampshire) piglets from four different litters were eligible for the study. Upon weaning, the piglets were randomly allocated either to a group fed a standard cereal based swine feed (hereafter referred to as Cereal group) supplemented with rapeseed oil in order to match fat intake in the two groups, or to a group fed a cereal free Paleolithic diet (hereafter referred to as Paleolithic group) consisting of vegetables, fruit, meat and a small amount of tubers. At baseline body weight did not differ between the groups (28.9 ± 2.9 kg vs. 30.7 ± 2.8 kg, Paleolithic vs. Cereal group, p = 0.2). In the Paleolithic group one pig, an apparent runt, failed to thrive from early post weaning and was culled at 3.5 months of age. Inclusion of meat (beef) was approved by the Swedish Board of Agriculture. Average intake during the study of protein, fat and carbohydrates were 17%, 18% and 65% respectively in the Cereal group, and 27%, 16% and 57% respectively in the Paleolithic group. Both diets were thus high in carbohydrate and low in fat compared to the spectrum of macronutrient intake estimated for contemporary hunter-gatherers [1]. For more detailed account of provisions during the last three months of the study, see Table 1. Both groups were fed their respective diet from 2 to 17 months of age by an experienced experimental pig farmer who allocated rations on a group basis judged sufficient to achieve healthy animals. Body weight was recorded every second week.

**Table 1:** Provisions during last three months in study

**Paleolithic group**	**kg/pig/day**	**kJ/pig/day**
Cabbage	1	915
Turnip	1	703
Cauliflower	0.7	661
Green pepper	0.05	34
Red pepper	0.05	55
Yellow pepper	0.05	47
Broccoli	0.15	179
Apple	1	2053
Pear	0.7	1286
Kiwi fruit	0.1	192
Water melon	0.1	149
Grape	0.03	88
Pineapple	0.03	60
Cherimoya	0.03	115
Potato	0.3	878
Carrot	0.3	474
Beetroot	0.1	160
Parsnip	0.05	106
Black radish	0.05	29
Beef	0.45	2995
Fish-meal	0.36	5882

**Total**	**6.6**	**17063**

		
**Cereal group**	**kg/pig/day**	**kJ/pig/day**

Cereal swine feed	1.5	18600
Rape-seed oil	0.06	2220

**Total**	**1.56**	**20820**

Energy intake at the end of the study was approximately 20% lower in the Paleolithic group as compared to the Cereal group despite much larger feed rations in terms of both volume and weight.

### Anaesthesia

At age 17 months all animals received premedication in the stable with intramuscular ketamine (Ketalar, Parke-Davis, Morris Plains, NJ), 7.5 mg/kg body weight and xylasin (Rompun, Bayer, Gothenburg, Sweden) 0.125 mg/kg. When sedated an intravenous needle was inserted in an ear vein and thiopental (Pentothal, Abbot, North Chicago, Il) 0.5 mg/kg and atropine (Atropine, Kabi Pharmacia, Uppsala, Sweden) 0.02 mg/kg were given. During the experiment, anesthesia was maintained using propofol (Diprivan, AstraZeneca, Södertälje, Sweden) 9–15 mg/kg/h. Fentanyl (Leptanal, Janssen-Cilag, Sollentuna, Sweden) 0.02 μg/kg and atracurium (Tracrium, Epipharm, Gnesta, Sweden) 0.2–0.5 mg/kg were given intermittently for pain management and muscular relaxation, respectively. The animals were ventilated with a Siemens Servo ventilator 300 (Siemens-Elema AB, Solna, Sweden). A volume-controlled, pressure-regulated ventilation of 14–20 L/min (15 breaths/min; positive end-expiratory pressure [PEEP], 5 cm H2O; inspired oxygen fraction, 0.5; max inspiratory pressure, 30 cm H_2_O) was used.

### Experimental protocol

An intravenous glucose tolerance test (IVGTT) was performed in 20 pigs which had been fasted for at least 6 hours. The pigs were kept in a supine position during the IVGTT, and hemodynamic values and body temperature (measured by naso-pharyngeal probe) were recorded using a data acquisition system (Testpoint; Capital Equipment Corp, Billerica, MA). A catheter was introduced into the external jugular vein and threaded into the superior vena cava for both glucose administration and blood sampling. At -5 min and -30 seconds before glucose administration, 2.5-ml blood samples were collected. At time 0, glucose (0.5 g/kg body weight) was infused over 20–30 seconds. Thereafter, 2.5 ml blood samples were drawn at 2, 3, 4, 5, 6, 8, 10, 12, 14, 16, 18, 19, 22, 25, 30, 35, 40, 45, 50, 55, 60, 75, 90, 105 and 120 min. An equivalent amount of sterile saline solution was used to flush the catheter line. The tubes were centrifuged, and plasma was stored at -20°C for analysis of insulin and glucose concentrations using standard methods. Body length was measured from nose to end of buttocks. Subcutaneous fat thickness was measured with a ruler at mid sternum after the thorax was opened. For immunohistochemistry of islet hormones and islet morphology, specimens were collected from mid- and tail portions of the pancreas from 5 pigs in each group. Two specimens were collected from each site. One specimen was fixed overnight in buffered formaldehyde (4 %), dehydrated in graded ethanols, and embedded in paraffin. The other specimen was fixed in Stefanini's solution (2 % paraformaldehyde and 0.2 % picric acid in 0.1 M phosphate buffer, pH 7.2), rinsed thoroughly in Tyrode solution containing 10 % sucrose, and frozen on dry ice. Sections from paraffin-embedded specimens, and cryosections were then processed for indirect immunofluorescence using well characterised islet hormone antisera as primary antibodies, and FITC-labelled second antibodies. For details on the antibodies used, and the immunostaining protocol, see e.g. Wierup et al 2002 [15].

### Data analysis and statistics

Continuous variables showed near Normal distribution in Normal plots (not shown), but in the case of C-reactive protein only after logarithmic transformation. Two pigs, one in each group, were obvious outliers given the extremely elevated insulin response to injected glucose (insulin area under the curve (AUC) 12 and 11 standard deviations above the group mean). These were excluded from calculations regarding glucose and insulin (basal and dynamic variables) and their respective correlations with other variables. Plasma glucose in remaining animals decreased exponentially (adjusted R^2 ^= 0.93 and 0.92 in the Paleolithic and Cereal group, respectively; p = 0.0001). Therefore, the glucose tolerance index K_G _could be calculated as the regression slope of the logarithmic transformation of glucose concentration versus time from 8 min, when problems related to glucose mixing are over. In contrast to glucose, which returned to values similar to pre-injection levels by 90 min (Figure 2), insulin remained elevated at 2 hours (Figure 3), precluding the use of the minimal model of glucose disappearance to estimate insulin sensitivity [16,17]. Fasting insulin sensitivity, which mostly accounts for the processes in the liver [18], was calculated with the QUICKI index [19]. For the dynamic part of the IVGTT, a surrogate of insulin sensitivity was calculated as glucose disappearance rate (expressed by K_G_) divided by the prevailing insulin (expressed by insulin AUC from 0 to 120 min (AUCins_0–120_)). Dynamic insulin sensitivity accounts for the insulin action at the level of muscle and adipose tissues [18]. Insulin secretion was evaluated as the suprabasal AUCins_0–120_, representing the glucose stimulated insulin response. Finally, acute insulin response was calculated as the average insulin concentration during the very early phase (2 to 4 min) after glucose injection. AUC was calculated with the trapezoidal rule. Data and results are expressed as mean ± standard deviation. Group comparisons of continuous variables were made by use of the unpaired two-sided Student's t-test with equal variances assumed. P < 0.05 was chosen for statistical significance. Bivariate correlations were evaluated by simple linear regression analyses, while multivariate correlations were evaluated by forward stepwise linear regression analyses. Due to the small sample size in this study only two variables were entered as independent variables in each regression model when analysing multivariate correlations, and even then the correlations should be interpreted with caution.

**Figure 1:**
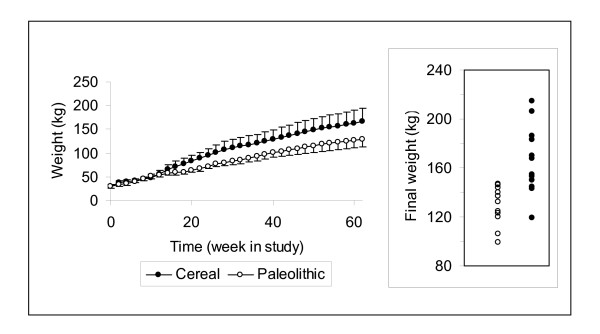
**Average weight during study and final weight**. The curves representing mean group weight started to diverge after 3 months of feeding the different diets. Spread of individual weights at the end of the study displayed some overlap between groups (insert).

**Figure 2:**
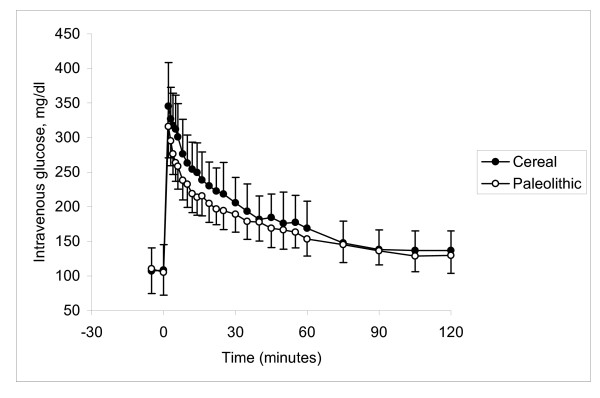
**Glucose response during glucose tolerance test**. No significant difference was observed between groups in average glucose response after an intravenous bolus of glucose (0.5 g/kg body weight) at age 17 months.

**Figure 3:**
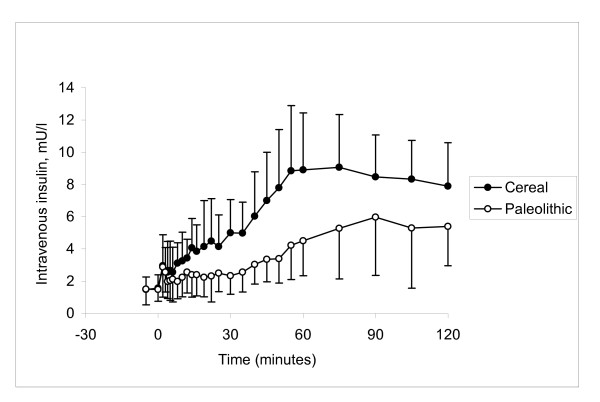
**Insulin response during glucose tolerance test**. Average insulin area under the curve from 0 to 120 minutes was significantly lower by 47% in the Paleolithic group as compared to the Cereal group after an intravenous bolus of glucose (0.5 g/kg body weight) at age 17 months (p = 0.001).

## Results

Energy intake at age 17 months was approximately 20% lower in the Paleolithic group despite much larger feed rations in terms of both volume and weight (Table 1). The mean weight curves for the two groups started to diverge after 3 months of feeding the different diets (Figure 1). At the end of the study the Paleolithic group weighed 22% less (129 ± 16 kg vs. 166 ± 28 kg, Paleolithic vs. Cereal, p = 0.0009; Table 2 and insert Figure 1) and was 6% shorter than the Cereal group (p = 0.003), while subcutaneous fat thickness was 43% lower (p = 0.0003; Table 2). No significant difference was seen in body temperature between groups (Table 2). The geometric mean of C-reactive protein was 82% lower in the Paleolithic group (p = 0.0007; Table 2). Intra-arterial diastolic blood pressure was significantly lower by 13% (p = 0.007) in the Paleolithic group, while systolic blood pressure was non-significantly lower by 7% (p = 0.12; Table 2).

**Table 2:** Final clinical characteristics (mean ± standard deviation)

	Paleolithic group (n = 11)	Cereal group (n = 12)	P
Weight (kg)	129 ± 16	166 ± 28	0.0009
Length (cm)	159 ± 6	170 ± 9	0.003
Subcutaneous fat (cm)	1.9 ± 0.4	3.3 ± 0.9	0.0003
Body temperature (°C)	37.7 ± 1.5	37.6 ± 0.5	0.8
CRP (μg/mL)^1^	4.0	21.7	0.0007
Systolic Blood Pressure (mm Hg)	140 ± 18	150 ± 9	0.12
Diastolic Blood Pressure (mm Hg)	108 ± 12	123 ± 12	0.007

^1 ^geometric mean, t-test on logarithmic CRP. Final clinical characteristics in pigs fed paleolithic or cereal diet from 2 to 17 months of age.

No significant difference was seen in mean fasting values of glucose or insulin between the groups (Table 3). Parameters related to the overall metabolic status indicated unchanged total glucose AUC (p = 0.14; Table 3, Figure 2) and glucose disappearance rate (K_G _= 0.58 ± 0.12 vs. 0.67 ± 0.17 %min^-1^, Paleolithic vs. Cereal, p = 0.20; Table 3). Fasting insulin sensitivity was not affected by diet (QUICKI = 0.66 ± 0.15 vs. 0.70 ± 0.36, Paleolithic vs. Cereal, p = 0.7; Table 3), but dynamic insulin sensitivity was markedly higher in the Paleolithic group (dynamic insulin sensitivity = 2.35 ± 0.76 vs. 1.41 ± 0.39 %min^-1^/(pmol/l), Paleolithic vs. control, p = 0.004; Table 3). Significant differences were also found in the insulin response to injected glucose (Table 3, Figure 3). AUCins_0–120 _was significantly lower in the Paleolithic group by 47% (p = 0.001) and stimulated insulin secretion was even more reduced by 58% (p = 0.0005). This reduction was mostly ascribed to the second phase insulin secretion, since the acute insulin response immediately following the glucose bolus (indicator of the early phase insulin release) was not different between the two groups (14.9 ± 8.3 vs. 15.8 ± 10.7 pmol/l, p = 0.8, Paleolithic vs. control; Table 3).

**Table 3:** Final glucometabolic characteristics (mean ± standard deviation)

	Paleolithic group (n = 9)	Cereal group (n = 9)	P
fP-glucose (mmol/l)	5.6 ± 1.4	6.0 ± 1.9	0.6
AUC^1 ^glucose 0–120 min (mmol/l min)	1076 ± 113	1199 ± 212	0.14
K_G _(%min-1)	0.58 ± 0.12	0.67 ± 0.17	0.20
fP-insulin (mmol/l)	8.3 ± 4.6	9.2 ± 4.6	0.7
QUICKI	0.66 ± 0.15	0.70 ± 0.36	0.7
Dynamic insulin sensitivity (%min-1/(pmol/l))	2.35 ± 0.76	1.41 ± 0.39	0.004
AUC^1 ^insulin 0–120 min (pmol/l min)	2613 ± 863	4 973 ± 1476	0.001
AUC^1 ^insulin 0–120 min stimulated secretion (pmol/l min)	1620 ± 1074	3872 ± 1112	0.0005
Acute insulin response 2–4 min (pmol/l)	14.9 ± 8.3	15.8 ± 10.7	0.8

^1^area under curve during intravenous glucose tolerance test. Final glucometabolic characteristics in pigs fed paleolithic or cereal diet from 2 to 17 months of age excluding cases fasting less than 6 hours.

Evaluations of bivariate correlation showed that weight was highly correlated with subcutaneous fat thickness (adjusted R^2 ^= 0.77, p < 0.0001). Evaluations of multivariate correlation with forward stepwise linear regression analysis was performed in order to study variables independently explaining the variation of dynamic insulin sensitivity, AUCins_0–120_, log C-reactive protein and diastolic blood pressure, respectively. In each of these four regression models diet was entered as independent variable and evaluated with each one of the remaining relevant study variables as the second independent variable. Diet emerged as the strongest explanatory variable for variations in dynamic insulin sensitivity, AUCins_0–120_, log C-reactive protein and diastolic blood pressure.

**Figure 4:**
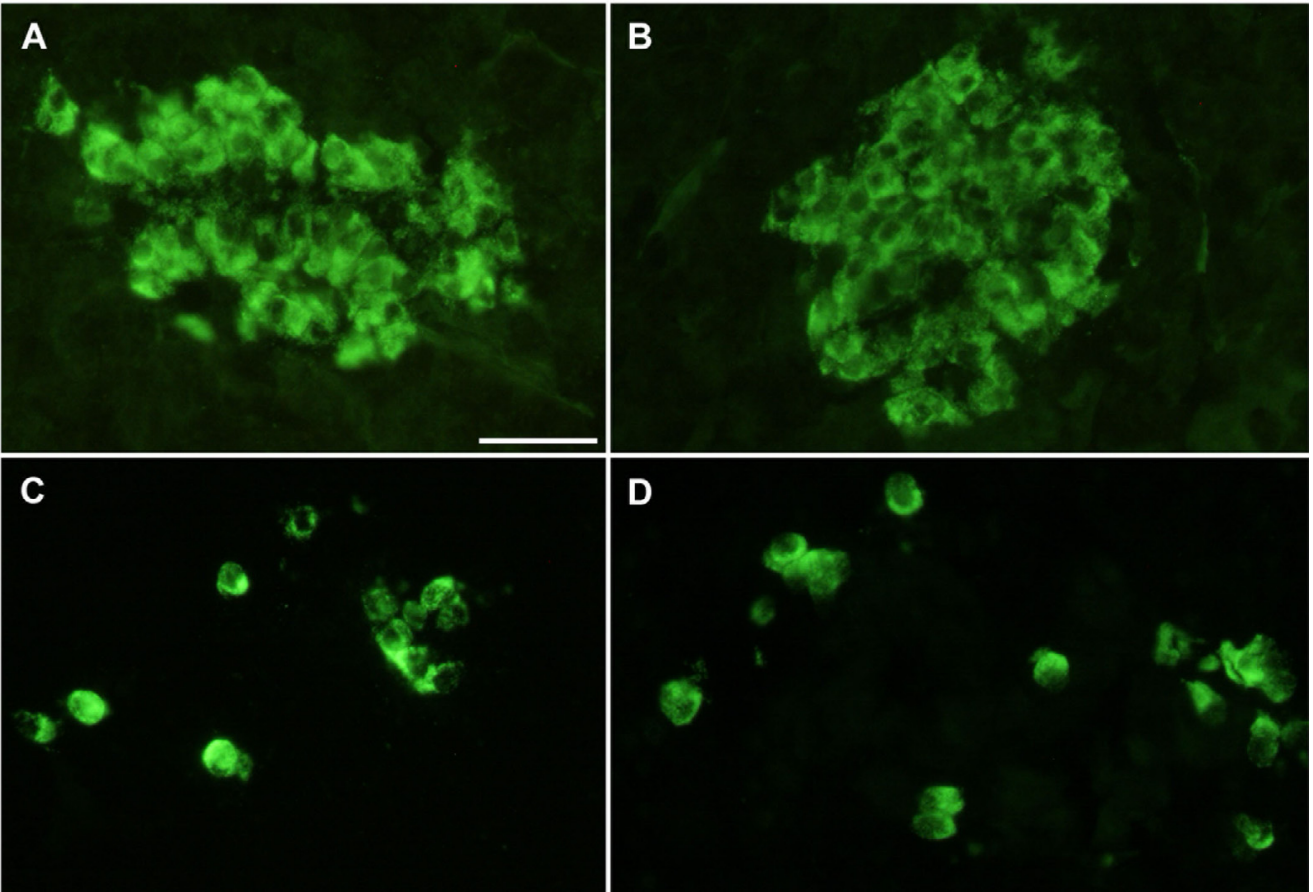
**Immunohistochemical staining of pancreatic islets**. Immunohistochemical staining of pancreatic islets showed no obvious difference between Cereal (A and C) and Paleolithic (B and D) groups regarding the frequencies or intraislet distribution patterns of cells storing insulin (A and B), glucagon (C and D), somatostatin and pancreatic polypeptide, nor was the islet size or islet frequency overtly different.

**Figure 5:**
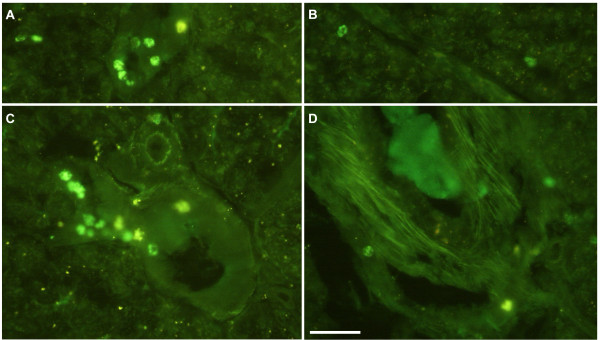
**Frequency of pancreatic leukocytes**. Leukocytes, as revealed by their unspecific binding of the secondary antibodies, their small size, and their characteristic nuclei, were clearly more frequent (more than doubled by cell counting) in all the sampled pigs from the Cereal group (A and C, n = 5) as compared to all the sampled pigs from the Paleolithic group (B and D, n = 5), with no overlapping between groups. These cells usually occurred scattered throughout the exocrine pancreatic parenchyma or clustered around pancreatic ducts (A and B) and blood vessels (C and D).

Immunohistochemical analysis comprised islet staining of insulin, glucagon, somatostatin and pancreatic polypeptide. There was no obvious difference between islets in the Cereal and Paleolithic groups regarding the frequencies or intra-islet distribution patterns of cells storing each of these four hormones, nor was the islet size or islet frequency overtly different (Figure 4). However, leukocytes, as revealed by their unspecific binding of the secondary antibodies, their small size, and their characteristic nuclei, were clearly more frequent (more than doubled by simple cell counting) in all the sampled pigs from the Cereal group as compared to all the sampled pigs from the Paleolithic group, with no overlapping between groups. These cells usually occurred scattered throughout the exocrine pancreatic parenchyma or clustered around pancreatic ducts and blood vessels (Figure 5).

## Discussion

This study showed highly beneficial effects of a Paleolithic diet on risk factors for diseases of affluence when compared to cereal based swine feed. A strength of the study is that the model used is one of the best non-primate models for human disorders, the domestic pig [20]. The present study indicated no difference in glucose disappearance during IVGTT between the groups. During IVGTT plasma insulin increased successively between 30 and 80 min in both groups, possibly due to propofol anesthesia [21,22], and this precluded the use of the minimal model of glucose disappearance [16,17]. However, other indices of dynamic insulin sensitivity clearly showed higher insulin sensitivity in the Paleolithic group, which also exhibited a significantly lower insulin response to injected glucose. It is worth noting that the difference in insulin response between groups during IVGTT was observed in the late phase of insulin secretion. Evaluation of multivariate correlation showed that the beneficial effect of Paleolithic diet on insulin sensitivity and insulin response was independent of all other relevant study variables such as body weight, subcutaneous fat thickness and body temperature. A Paleolithic diet thus conferred higher insulin sensitivity, which is central to the prevention of cardiovascular disorders [23], and consistent with our finding in humans [24]. Furthermore, contrary to our survey in humans [24], this finding of higher insulin sensitivity was associated with no significant difference between groups in fasting levels of insulin and glucose, indicating that the Paleolithic diet affected insulin action mainly at the level of muscle and adipose tissues rather than the liver [18].

The Paleolithic group also showed significantly lower levels of C-reactive protein, a physiologic marker of subclinical inflammation, which has been shown to be associated with insulin resistance and cardiovascular disease [23,25]. Elevated levels of C-reactive protein has been suggested to reflect overproduction by expanded adipose tissue mass [23]. However, evaluation of multivariate correlation showed that the beneficial effect of Paleolithic diet on C-reactive protein was independent of all other relevant study variables including measures of obesity such as body weight and subcutaneous fat thickness. Interestingly, immunohistochemical analysis suggested a diffuse and low-grade pancreatic inflammation in the cereal group, as evidenced by clearly more frequent leukocytes scattered throughout their exocrine pancreatic parenchyma or clustered around pancreatic ducts and blood vessels. This finding offers a novel approach in the research on the association between inflammation and type 2 diabetes [25]. Immunohistochemical analysis also showed that the cereal group could compensate for both pancreatic inflammation and increased need of insulin due to lower insulin sensitivity, without noticeable differences in pancreatic islets between groups.

The shorter length in the Paleolithic group is not unexpected if we consider length in pigs as a correlate of height in humans. Available evidence suggests that hunter-gatherers and similar ethnic groups are shorter than Western populations, and a positive relationship between height and cardiovascular disease has been noted in international comparisons [26]. Insulin resistance and hyperinsulinemia can hypothetically promote growth by way of insulin-like growth factors [27].

In addition to these significant results, intra-arterial diastolic blood pressure was significantly lower in the Paleolithic group, and this beneficial effect of Paleolithic diet was also independent of all other relevant study variables. The present study thus provides experimental evidence for beneficial effects of a Paleolithic diet on risk factors for diseases of affluence, which might account for their low incidence reported among Non-Western ethnic groups with hunter-gatherer lifestyles and diets [6,7].

### Effecting mechanisms

The mechanisms behind these beneficial effects on risk factors are largely unknown. In evaluations of multivariate correlation, diet emerged as a stronger explanatory variable than any other relevant variable for variations in dynamic insulin sensitivity, AUCins_0–120_, log C-reactive protein and diastolic blood pressure. The observed effects on risk factors thus seem to be primarily caused by diet. However, it is conceivable that this dietary effect on risk factors is a result of differences in other variables induced by dietary assignment. An important finding in this regard is the divergence of the weight curves between the two groups after 3 months of feeding. The Paleolithic group was thus lighter and had a lower energy intake at the end of the study than the Cereal group, despite a threefold larger ration by weight. The diverging weight curves could be interpreted in several ways. They could be interpreted as food restriction in the Paleolithic group, which could explain the observed effects on risk factors [28]. Neither group was fed their respective diet *ad libitum *but was rather allocated rations on a group basis judged sufficient to achieve healthy animals by an experienced experimental pig farmer. This procedure was chosen due to concerns about the unfamiliar Paleolithic diet as swine feed, and the fact that feeding *ad libitum *is not the custom in Swedish swine production. The diverging weight curves between the groups could thus possibly be caused by differences in subjectively allocated rations (e.g. difference in energy intake), which could lead to food restriction in the Paleolithic group. However, food restriction typically lowers mean body temperature by 1–2°C [29], and we found no significant difference in mean body temperature between the two groups, indicating that there was not a substantial food restriction in the Paleolithic group as compared to the Cereal group. In fact, the mean weight in the Paleolithic group is well within the normal range of pigs [30]. The diverging weight curves are thus probably not caused by food restriction in the Paleolithic group but instead could be interpreted as obesity in the Cereal group in analogy with human classification of individual weight based on statistical health effects [31]. The significant difference in subcutaneous fat thickness between groups and the high correlation between weight and subcutaneous fat thickness supports this notion. Obesity in humans is associated with increased insulin resistance, high blood pressure and high levels of C-reactive protein [23], and could explain the observed effects on risk factors. The difference between the two diets regarding obesity promotion could still be due to differences in subjectively allocated rations, but could alternatively be due to properties of a cereal based swine feed which possibly disturb the regulation of satiation [3], satiating properties of a Paleolithic diet [2] and differing effects between the diets on energy metabolism [2]. The satiating properties of a Paleolithic diet could be due to differences in macronutrient diet composition, such as the low protein content of cereals in the Cereal group diet. The Paleolithic group thus ate relatively more protein and less carbohydrates, which through satiating, thermogenic and other properties could account for the results of the study [32,33], although results from studies on the association between protein intake and diseases of affluence have been contradictory [34]. In future studies of Paleolithic diets it would be valuable to match diets for macronutrient composition. The latter alternative explanations for the diverging weight curves is supported by our epidemiological findings in humans from Kitava, Papua New Guinea, where a non-western lifestyle has been connected to leanness despite food being available *ad libitum *[13]. The diverging weight curves would then suggest that the beneficial effects on risk factors could be due to differences between diets regarding obesity promotion. Alternatively, obesity could be a marker for other effecting mechanisms, such as leptin resistance, and the diverging weight curves would then suggest that the beneficial effects on risk factors could be due to differences between diets regarding promotion of leptin resistance [3].

## Conclusion

In conclusion, this study in domestic pigs suggests that a Paleolithic diet as compared to a cereal based diet conferred higher insulin sensitivity, lower C-reactive protein and lower blood pressure. The results suggest that pigs, as previously suggested for humans [3], are not specifically adapted through evolution to a diet incorporating large amounts of cereals, which could confer diseases of affluence as a sign of insufficient evolutionary adaptation [4].

## Competing interests

The author(s) declare that they have no competing interests.

## Authors' contributions

TJ participated in the design of the study and in carrying out the experiments at the end of the study, participated in statistical analysis, and conceived of and wrote the article. BA participated in the design of the study, carried out the analysis of glucose and insulin, and conceived of and participated in the design of the article as well as revising it for important intellectual content. GP participated in the statistical analysis of glucose and insulin and revised the article for important intellectual content. FS and NW participated in carrying out the experiments at the end of the study, and carried out the immunohistochemical studies. SS and TS participated in the overall design and coordination of the study, were responsible for the animals and the laboratory where the experiment was performed, and participated in carrying out the experiments at the end of the study. MU participated in carrying out the experiments at the end of the study and revised the article for important intellectual content. JF carried out the analysis on C-reactive protein and revised the article for important intellectual content. LG revised the article for important intellectual content. SL conceived of and participated in the design, coordination and execution of the study, participated in carrying out the experiments at the end of the study, participated in statistical analysis, conceived of and participated in the design of the article as well as revising it for important intellectual content. All authors read and approved the final manuscript.
